# Changes in facial width according to the ostectomy level of the proximal bone segment in intraoral vertical ramus osteotomy for mandibular prognathism

**DOI:** 10.1186/s40902-022-00347-5

**Published:** 2022-04-18

**Authors:** Sang-Hoon Kang, Min-Jun Kang, Min-Ji Kim, Moon-Key Kim

**Affiliations:** Department of Oral and Maxillofacial Surgery, National Health Insurance Service Ilsan Hospital, 100 Ilsan-ro, Ilsan-donggu, Goyang, Gyeonggi-do 10444 Republic of Korea

**Keywords:** Proximal segment ostectomy, Intraoral vertical ramus osteotomy, Mandibular prognathism, Facial width

## Abstract

**Background:**

This study aimed to investigate the changes in facial width according to the ostectomy level of the proximal segment after orthognathic surgery using intraoral vertical ramus osteotomy (IVRO) in patients with mandibular prognathism.

**Methods:**

The participants included 32 individuals who were diagnosed with class III malocclusion prior to surgery. All participants underwent orthognathic surgery using either version of IVRO. The surgery patients were categorized into two groups depending on the type of proximal bone-segment ostectomy technique used: patients whose osteotomy height was at the level of the mandibular tooth occlusal surface (the mandibular tooth surface–level group) and patients whose osteotomy height was at the level of the mandibular inferior border (the mandibular inferior border–level group). The distances between the mandibular width and soft tissue width at the height of the sigmoid notch, mandibular foramen, and alveolar bone and at the anterior-posterior location of the mandibular condyle, mandibular foramen, and coronoid process were compared between the groups. All data were compared to identify differences between preoperative and postoperative measurements.

**Results:**

The postoperative change in facial soft tissue width at the intersection of the coronal plane with the coronoid process and the horizontal plane at the height of the mandibular alveolar bone in the group with osteotomy at the level of the mandibular occlusal surface differed significantly from that in the group with osteotomy at the level of the mandibular inferior border, with respective increases (mean ± SD) of 1.3 ± 3.5% and 4.7 ± 5.6%, compared to preoperative measurements (*p* = 0.050).

**Conclusions:**

Proximal segment ostectomy at the level of the mandibular occlusal surface must be considered with regard to postoperative facial soft tissue width in vertical ramus osteotomy. Additionally, it is necessary to study the visual effect of the width of the mandible appearing small because of the posterior position of the mandible, even when the mandibular facial width is maintained.

## Background

Orthognathic surgeries that are more commonly used to treat mandibular prognathism include intraoral vertical ramus osteotomy (IVRO) and sagittal split ramus osteotomy (SSRO). IVRO involves vertical osteotomy of the ramus of the mandible posterior to the mandibular foramen and subsequent mandibular setback by overlapping the mandibular bone segments, resulting in an increase in mandibular width [1, 2].

The process of osseous healing is similar for both SSRO and IVRO [3]. A long-term follow-up study on changes in facial width after surgery for mandibular prognathism reported that IVRO did not promote an increase in facial width [4].

IVRO requires an additional partial ostectomy of the proximal bone to adjust the length of the proximal bone segment to the distal mandibular bone ramus length after mandibular ramus osteotomy. To our knowledge, no study has investigated the changes in facial width in relation to the degree of ostectomy of the proximal bone segment during IVRO.

Recently, a common surgical approach has been to first perform preoperative orthognathic simulation surgery through analysis of the three-dimensional (3D) skeletal structure using maxillofacial computed tomography (CT) imaging and then perform the actual surgery based on this information [5]. The use of three-dimensional imaging allows researchers to conduct a more detailed facial measurement study of changes in facial width after ostectomy of the proximal bone segment in IVRO.

This study aimed to investigate changes in facial mandibular width in relation to the degree of ostectomy of the proximal bone segment in patients who underwent orthognathic IVRO mandibular setback surgery for mandibular prognathism.

## Materials and methods

The participants included patients diagnosed with class III malocclusion prior to surgery. The inclusion criteria were patients who underwent orthognathic surgery using IVRO, had a complete medical record with CT images, and did not have prior mandibular setback surgery. Patients were considered to have CT images when pre-, intra-, and post-orthognathic surgery CT images were obtained.

The exclusion criteria were as follows: patients who underwent other mandibular ramus surgical procedures such as SSRO, subcondylar osteotomy, and L-shaped ramus osteotomy; those who had missing CT images more than 1 year post-surgery; those who did not have preoperative three-dimensional analysis; those whose CT images were not retrieved; those who were diagnosed at different hospitals and received referral for orthognathic surgery at our hospital; and those whose surgical records lacked details. Only CT images with a slice thickness <1 mm were used in this study. In addition, patients with lesions causing changes in the mandible were excluded from this study. IVRO was defined as vertical osteotomy of the ramus along with partial ostectomy of the proximal segment of the mandibular ramus.

**Fig. 1:**
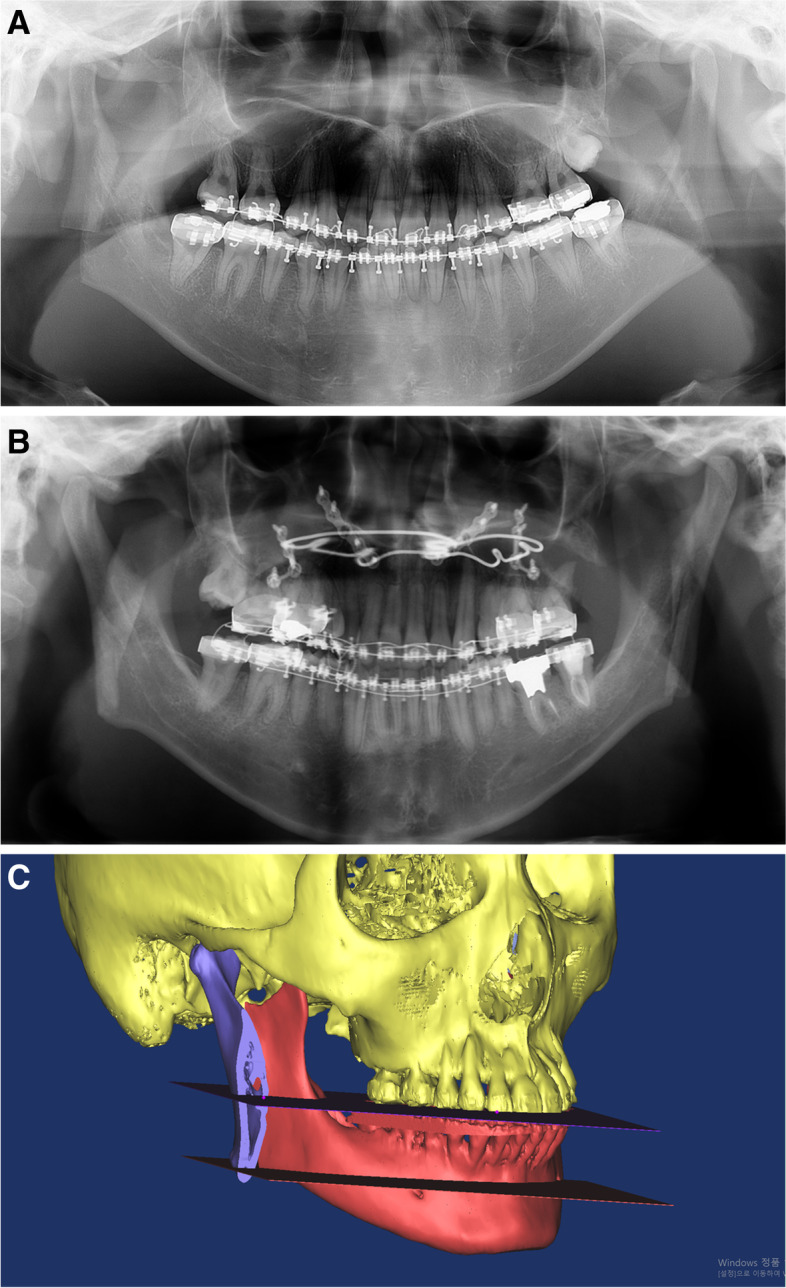
For example, panoramic radiographs and three-dimension (3D) image for the osteotomy group. The surgery patients were categorized into two groups depending on the level of the mandibular ostectomy used on the proximal segment. **A** Mandibular tooth surface–level group (superior ostectomy group; SO group) comprised patients in whom the height of the ostectomy of the proximal bone segment was at the level of the mandibular tooth occlusal surface. **B** Mandibular inferior border–level group (inferior ostectomy group; IO group) comprised patients in whom the height of the mandibular osteotomy of the proximal bone segment was at the level of the mandibular inferior border cortex. **C** Three-dimensional image for the mandibular tooth surface–level group (superior ostectomy group; SO group) and mandibular inferior border–level group (inferior ostectomy group; IO group). The two horizontal planes show the cut heights of the proximal segment. The upper and lower planes correspond to the SO and IO groups, respectively

The surgical patients were categorized into two groups depending on the type of mandibular ostectomy level used on the proximal segment (Fig. 1). The mandibular tooth surface–level group (superior ostectomy group; SO group) comprised patients whose ostectomy height of the proximal bone segment was at the level of the mandibular tooth occlusal surface. The mandibular inferior border–level group (inferior ostectomy group IO group) comprised patients whose ostectomy height of the proximal bone segment was at the level of the mandibular inferior border cortex. During IVRO, the height of the proximal segment of the ramus to be osteotomized was determined. When cutting at the height of the tooth surface, the reciprocating saw should match the occlusal surface of the teeth to cut the proximal segment of the ramus. When cutting at the level of the inferior border of the mandible, the blade of the saw should be oriented the same as the lower edge of the mandible for cutting the proximal segment.

The preoperative and 1-year postoperative CT images were compared.

This retrospective study design complied with and was approved by the institutional review board (No. NHIMC-2021-02-017).

### Measurement method

Simplant 14.0 ver. (Materialise NV, Leuven, Belgium) was used to reconstruct the CT images and obtain 3D images of the facial bones and soft tissues. Each anatomical reference point on the facial soft tissue surface area of the mandible was determined for facial soft tissue measurements (Tables 1 and 2). In setting the reference planes, reference points, such as the nasion, porion, orbitale, and foramen magnum, were marked on the facial skeleton. The specific measurements used in this calculation, including the points and planes, were as follows:

**Table 1:** Description of the points

Point	Description
Nasion	The middle point of frontonasal suture
Porion	The upper margin of ear canal
Orbitale	The lowest point on the infraorbital margin
Menton	The lowest point on the mandibular symphysis
Mandibular F	Mandibular foramen
Coronoid pr	The tip of the coronoid process
Sigmoid N	The lowest point of the sigmoid notch
Condyle	The most superior point of the condyle head
#31-#41	Midpoint between the mesial incisal tips of the both mandibular central incisors
#36 cervical	Buccal alveolar bone margin of the left mandibular first molar
#46 cervical	Buccal alveolar bone margin of the right mandibular first molar
B: MnF-C/Mn F-H	Outermost bone point where Mandibular F Coronal plane and Mandibular F plane intersect
B: MnF-C/Alv-H	Outermost bone point where Mandibular F Coronal plane and Alveolar plane intersect
ST: condyle-C/Sig N-H	Outermost soft tissue point where Condyle Coronal plane and Sigmoid N plane intersect
ST: condyle-C/Mn F-H	Outermost soft tissue point where Condyle Coronal plane and Mandibular F plane intersect
ST: condyle-C/Alv-H	Outermost soft tissue point where Condyle Coronal plane and Alveolar plane intersect
ST: MnF-C/Sig N-H	Outermost soft tissue point where Mandibular F Coronal plane and Sigmoid N plane intersect
ST: MnF-C/Mn F-H	Outermost soft tissue point where Mandibular F Coronal plane and Mandibular F plane intersect
ST: MnF-C/Alv-H	Outermost soft tissue point where Mandibular F Coronal plane and Alveolar plane intersect
ST: coronoid C/Sig N-H	Outermost soft tissue point where Coronoid pr Coronal plane and Sigmoid N plane intersect
ST: coronoid C/Mn F-H	Outermost soft tissue point where Coronoid pr Coronal plane and Mandibular F plane intersect
ST: coronoid C/Alv-H	Outermost soft tissue point where Coronoid pr Coronal plane and Alveolar plane intersect

**Table 2:** Description of the planes

	Name	Description
Horizontal	FH plane	Plane defined by point Porion left, point Porion right and point Orbitale-Mid
	Sigmoid N plane	Plane through point Sigmoid N-Mid and parallel to plane FH plane
	Mandibular F plane	Plane through point Mn Foramen-Mid and parallel to plane FH plane
	Alveolar plane	Plane through point #36-46 cervical-Mid and parallel to plane FH plane
Vertical	Porion Coronal plane	Plane through point Porion left and Porion right and normal to plane FH plane
	Condyle Coronal plane	Plane through point Condyle-left and Condyle-right and normal to plane FH plane
	Mandibular F Coronal plane	Plane through point Mandibular F-left and Mandibular F-right and normal to plane FH plane
	Coronoid pr Coronal plane	Plane through point Coronoid pr-left and Coronoid pr-right and normal to plane FH plane

Preoperative and postoperative CT images were imported into the Simplant software. 3D images were constructed, and measurements were obtained using both three-dimensional preoperative and postoperative CT data. Two basic reference planes were constructed. The basic horizontal plane was used to construct the Frankfort horizontal (FH) plane for the measurement of vertical error. The FH plane intersects the midpoint of the bilateral orbitale points on the infraorbital margin and the two porion points on the bilateral external auditory canals. The basic coronal plane was defined as the porion coronal plane (Porion Coronal plane), which is perpendicular to the FH plane and crosses the bilateral porion points.

### Reference points

The mandibular foramen (Mandibular F), coronoid process (Coronoid pr), sigmoid notch (Sigmoid N), condyle (Condyle), menton (Menton), and bilateral superior buccal alveolar bone ridge of the first molar (#36 cervical, #46 cervical) were placed on the mandibular bone. Subsequently, the most external mandibular bone and the most external facial soft tissue points on each horizontal and coronal plane (see definitions in the “Reference planes” section) that intersect were set as the measurement points for the width. In addition, the facial soft tissue points were marked to represent the measurement points for facial width on the epidermal layer of the facial soft tissue on the horizontal and coronal planes (Table 1).

### Reference planes

**Fig. 2:**
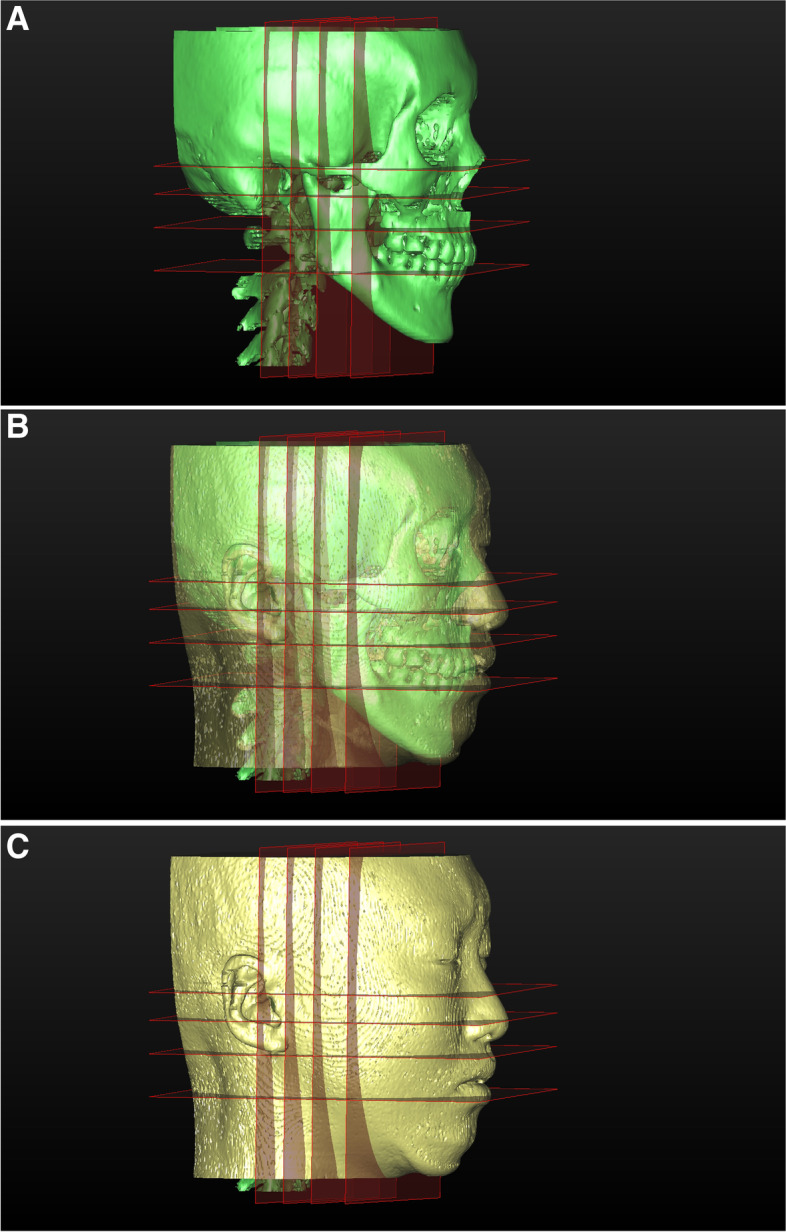
The horizontal and coronal planes. From top to bottom, the FH plane used as the basic horizontal plane and the horizontal planes for measurement: the Sigmoid N plane, Mandibular F plane, and Alveolar plane. From medial to distal, the Porion Coronal plane used as the basic coronal plane and the coronal planes for measurement: the Condyle Coronal plane, Mandibular F Coronal plane, and Coronoid pr Coronal plane. **A** Facial skeletal image and planes. **B** Combined image with facial skeletal and soft tissue image. **C** Facial soft tissue image and planes

The horizontal planes were defined as planes parallel to the FH plane, which was the basic horizontal plane. The coronal planes were defined as planes parallel to the Porion Coronal plane, which was the basic coronal plane (Table 2, Fig. 2). The horizontal planes were characterized by reference points, including the sigmoid notch, mandibular foramen, and first molar alveolar bone, according to their heights on the mandible. The horizontal planes were the Sigmoid N plane, Mandibular F plane, and Alveolar plane (Fig. 2). The coronal planes were characterized by reference points, including the condyle, mandibular foramen, and coronoid process, according to their anteroposterior positions on the mandible. The coronal planes were the Condyle Coronal plane, Mandibular F Coronal plane, and Coronoid pr Coronal plane (Fig. 2).

### Measurement item definition

The mandibular bone (B width) and soft tissue (ST width) measurements are shown in Table 3.

**Table 3:** Description of measurements

Name	Descriptions
B Width MnF-C/Mn F-H	Distance between point Left B: MnF-C/Mn F-H and point Right B: MnF-C/Mn F-H
B Width MnF-C/Alv-H	Distance between point Left B: MnF-C/Alv-H and point Right B: MnF-C/Alv-H
ST Width condyle-C/Sig N-H	Distance between point Left ST: condyle-C/Sig N-H and point Right ST: condyle-C/Sig N-H
ST Width condyle-C/Mn F-H	Distance between point Left ST: condyle-C/Mn F-H and point Right ST: condyle-C/Mn F-H
ST Width condyle-C/Alv-H	Distance between point Left ST: condyle-C/Alv-H and point Right ST: condyle-C/Alv-H
ST Width MnF-C/Sig N-H	Distance between point Left ST: MnF-C/Sig N-H and point Right ST: MnF-C/Sig N-H
ST Width MnF-C/Mn F-H	Distance between point Left ST: MnF-C/Mn F-H and point Right ST: MnF-C/Mn F-H
ST Width MnF-C/Alv-H	Distance between point Left ST: MnF-C/Alv-H and point Right ST: MnF-C/Alv-H
ST Width coronoid C/Sig N-H	Distance between point Left ST: coronoid C/Sig N-H and point Right ST: coronoid C/Sig N-H
ST Width coronoid C/Mn F-H	Distance between point Left ST: coronoid C/Mn F-H and point Right ST: coronoid C/Mn F-H
ST Width coronoid C/Alv-H	Distance between point Left ST: coronoid C/Alv-H and point Right ST: coronoid C/Alv-H

B Width of MnF-C/Mn F-H: Distance between point Left B: MnF-C/Mn F-H and point Right B: MnF-C/Mn F-H

B Width MnF-C/Alv-H: Distance between points Left B: MnF-C/Alv-H and Right B: MnF-C/Alv-H

ST Width condyle-C/SigN-H: Distance between points Left ST: condyle-C/SigN-H and point Right ST: condyle-C/SigN-H

ST Width condyle-C/Mn F-H: Distance between points Left ST: condyle-C/Mn F-H and point Right ST: condyle-C/Mn F-H

ST Width condyle-C/Alv-H: Distance between points Left ST: condyle-C/Alv-H and point Right ST: condyle-C/Alv-H

ST Width MnF-C/SigN-H, ST Width MnF-C/Mn F-H, ST Width MnF-C/Alv-H, ST Width coronoid C/SigN-H, ST Width coronoid C/Mn F-H, and ST Width coronoid C/Alv-H were measured based on the above definitions. The reference points are changed according to the reference planes, but the measurement method is the same.

Widths were defined as the horizontal distance between the lateral point of the mandible and facial soft tissue and the points at which each horizontal and coronal plane intersected (Table 1, Fig. 2). Mandibular setback distance refers to the vertical distance between the mandibular chin Menton point and the posterior coronal plane.

### Statistical analysis

The mandibular width and soft tissue width of the distances between each bilateral pair of external points at each horizontal plane and coronal plane were compared between the groups. All data were compared to identify the differences between the preoperative and postoperative measurements (calculation: postoperative value minus preoperative value) using a *t*-test. In addition, the differences between the preoperative and postoperative measurements (calculation: postoperative value minus the preoperative value) were compared with the preoperative measurements to calculate the comparative percentage change using a *t*-test. Data were analyzed using IBM SPSS Statistics 23 (IBM, Armonk, New York, USA). The significance level was set at *p* ≤ 0.05.

## Results

Of all the participants who met the inclusion criteria for the study of different proximal segment osteotomy techniques during IVRO, 22 constituted the superior ostectomy group (SO group), who underwent superior proximal segment osteotomy at the level of the mandibular tooth occlusal surface, and 10 constituted the inferior ostectomy group (IO group) who underwent inferior proximal segment osteotomy at the level of the mandibular inferior border cortex. There were no significant differences between the males and females in the two groups. The average ages (mean ± SD) of the participants in the SO and IO groups were 21.1 ± 3.3 years and 19.7 ± 2.6 years, respectively.

In the case of ST Width coronoid C/Alv-H, the postoperative change in facial soft tissue width (mean ± SD % compared with the preoperative measurement) in the SO group was 1.3 ± 3.5% and statistically significantly less than the 4.7 ± 5.6% change in the IO group (*p* = 0.050) (Table 4).

**Table 4:** Postoperative change in facial width (as a percentage of the preoperative width) according to the ostectomy level

	Superior ostectomy group (*n* = 22)	Inferior ostectomy group (*n* = 10)	*P*-value
B Width MnF-C/Mn F-H	5.7±4.4	4.6±5.6	0.566
B Width MnF-C/Alv-H	0.9±3.5	1.9±4.7	0.502
ST Width condyle-C/Sig N-H	1.1±2.5	1.9±2.2	0.359
ST Width condyle-C/Mn F-H	2.5±4.2	3.6±3.2	0.454
ST Width condyle-C/Alv-H	0.3±5.6	4.0±6.1	0.101
ST Width MnF-C/Sig N-H	1.4±2.4	2.5±2.4	0.231
ST Width MnF-C/Mn F-H	2.0±3.0	4.4±4.9	0.099
ST Width MnF-C/Alv-H	0.4±4.8	3.5±5.7	0.124
ST Width coronoid C/Sig N-H	1.9±2.0	1.7±2.9	0.801
ST Width coronoid C/Mn F-H	2.4±2.6	3.8±3.8	0.205
ST Width coronoid C/Alv-H	1.3±3.5	4.7±5.6	0.050*

Superior ostectomy group: patient group with proximal segment ostectomy on mandibular tooth surface level

Inferior ostectomy group: patient group with proximal segment ostectomy on mandibular inferior border level

*P ≤ .05

The postoperative ST Width coronoid C/Alv-H in the SO group increased over the preoperative width by 1.6 ± 4.2 mm and width in the IO group increased over the preoperative width by 5.6 ± 7.1 mm (*p* = 0.053) (Table 5).

**Table 5:** Postoperative change in facial width (mm) after IVRO according to the proximal segment ostectomy level

	Superior ostectomy group (*n*=22)	Inferior ostectomy group (*n*=10)	*P*-value
B Width MnF-C/Mn F-H	5.8±4.5	4.5±5.6	0.507
B Width MnF-C/Alv-H	0.9±3.5	1.9±4.7	0.529
ST Width condyle-C/Sig N-H	1.6±3.7	2.9±3.4	0.341
ST Width condyle-C/Mn F-H	3.4±6.1	5.3±4.8	0.405
ST Width condyle-C/Alv-H	0.1±7.1	5.3±8.5	0.079
ST Width MnF-C/Sig N-H	2.0±3.5	3.8±3.8	0.195
ST Width MnF-C/Mn F-H	2.8±4.3	6.3±7.3	0.099
ST Width MnF-C/Alv-H	0.6±5.9	4.6±7.8	0.123
ST Width coronoid C/Sig N-H	2.8±3.0	2.5±4.3	0.824
ST Width coronoid C/Mn F-H	3.2±3.5	5.3±5.5	0.204
ST Width coronoid C/Alv-H	1.6±4.2	5.6±7.1	0.053

Superior ostectomy group: patient group with proximal segment ostectomy on mandibular tooth surface level

Inferior ostectomy group: patient group with proximal segment ostectomy on mandibular inferior border level

In the case of B Width MnF-C/Mn F-H, the differences in facial widths in the SO group and the IO group (mean ± SD) were 5.8 ± 4.5 mm and 4.5 ± 5.6 mm, respectively, and compared with the preoperative measurement, they did not differ significantly (*p* = 0.507) (Table 5).

All facial widths and measurement ratios, excluding the ST Width coronoid C/Alv-H, did not differ significantly between the IO and SO groups.

A mandibular setback was found in both groups postoperatively. On the Porion Coronal plane, the vertical distance (mean ± SD) between the reference and the Menton point was reduced to 8.4 ± 3.8 mm in the SO group and to 7.3 ± 2.3 mm in the IO group (*p* = 0.287).

## Discussion

When viewed on the Coronoid pr Coronal plane and Alveolar plane at the height of the mandibular first molar alveolar bone, the increase in facial soft tissue width compared with the preoperative width was statistically significantly smaller in the SO group (1.3%) than in the IO group (4.7%). The facial width increased by 1.6 mm on average in the SO group, whereas it increased by 5.6 mm in the IO group. The facial widths and measurement ratios showed no significant differences between the two different post-osteotomy proximal segment treatment groups, except for the ST Width coronoid C/Alv-H.

**Fig. 3:**
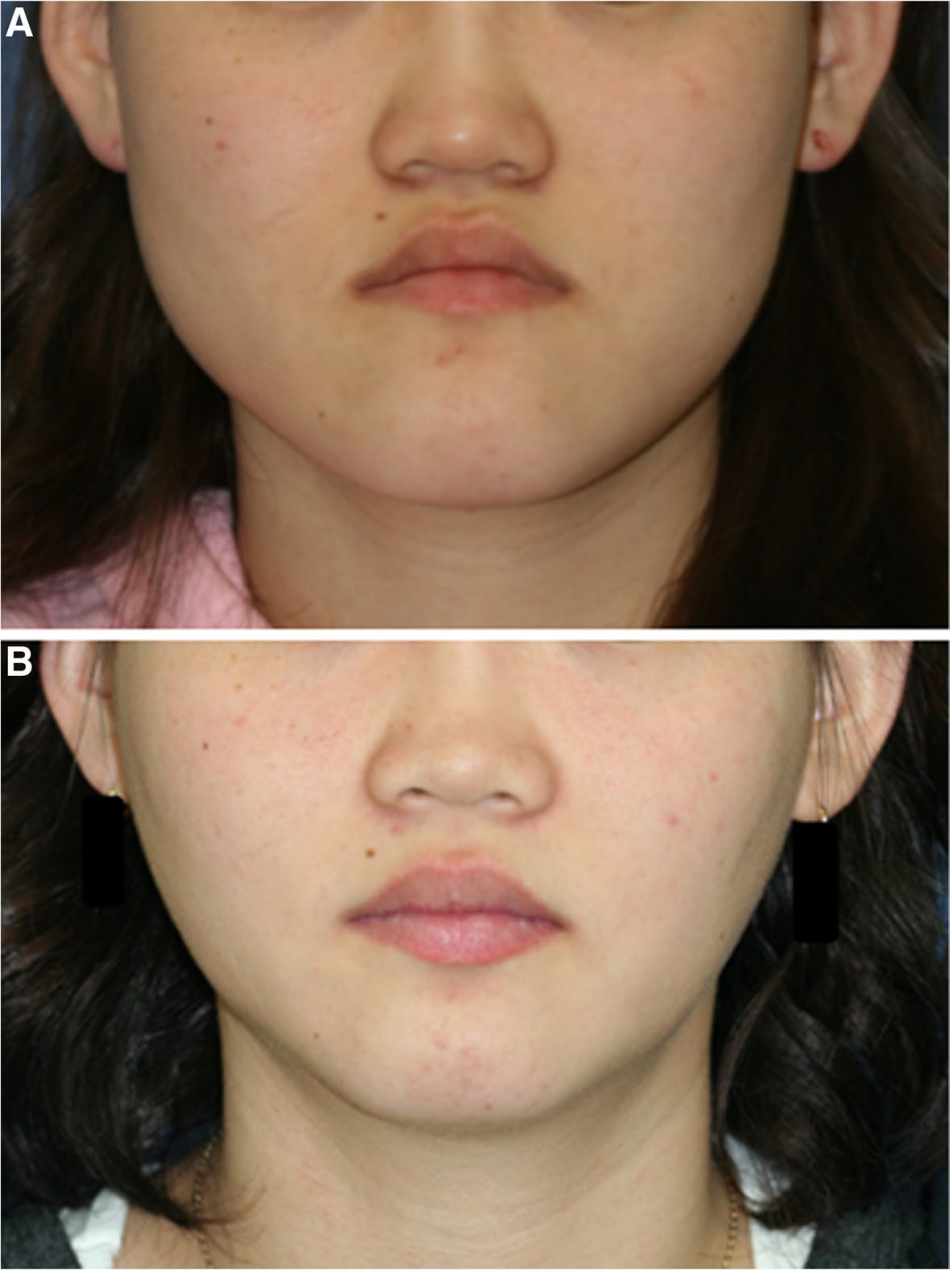
Clinical photography of the patient in the superior ostectomy group. **A** Photograph before the orthognathic surgery. **B** Photograph 7 months after the postoperative orthodontic treatment

**Fig. 4:**
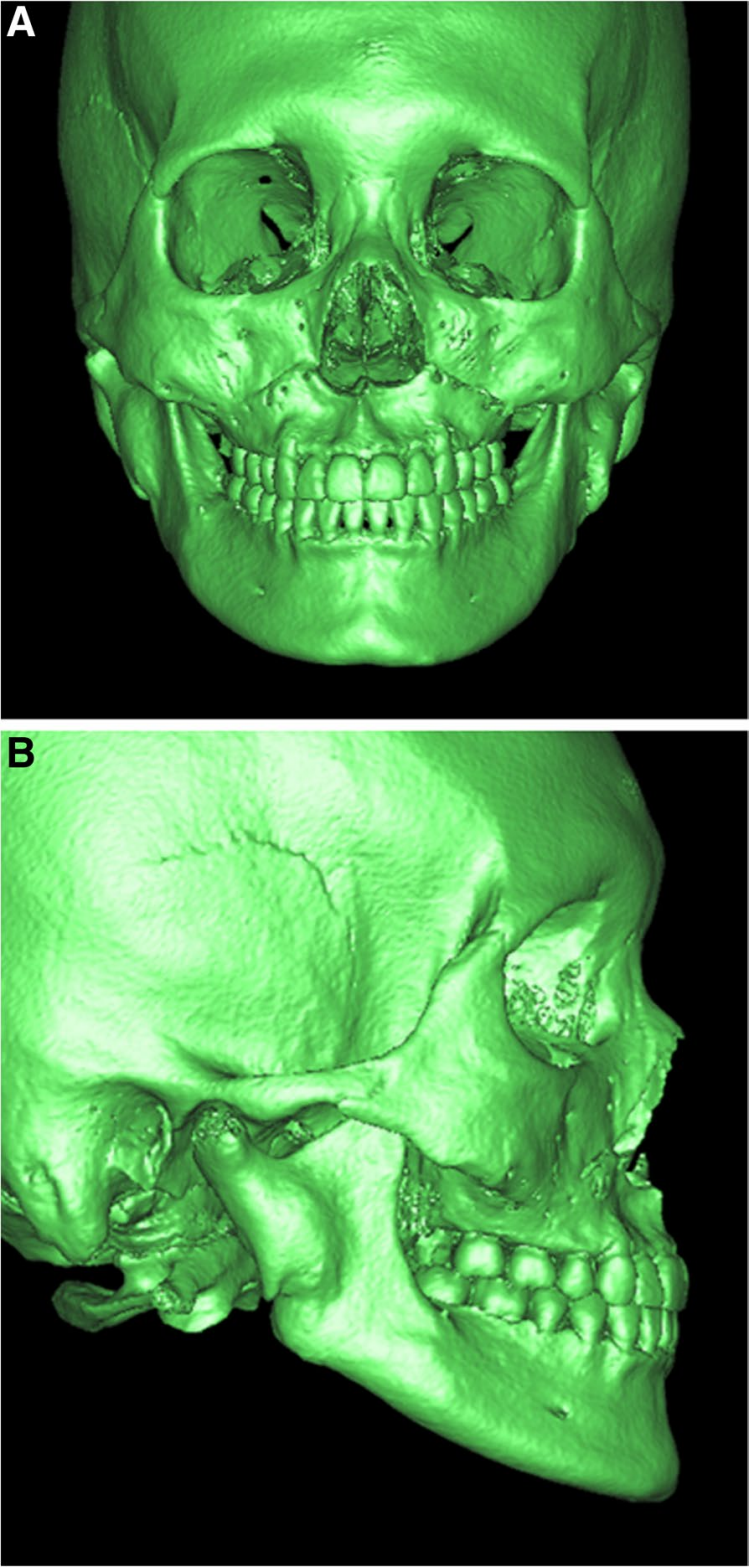
Three-dimensional CT images of the patient 7 months after the postoperative orthodontic treatment in the superior ostectomy group. **A** Anteroposterior view. **B** Lateral view

The results of this study suggest that the choice of the proximal segment ostectomy procedure in IVRO affects the postoperative facial width. There were clinical photographs of one patient before and after the surgery (Fig. 3) and 3D CT images of the patient 7 months after the postoperative orthodontic treatment in the superior ostectomy group (Fig. 4). However, it is difficult to accurately predict the movement of the proximal segment from the TMJ and the bone-healing progress after IVRO. In this study, the measurement point near the ramus osteotomy area could not be accurately defined, thus limiting the measurement of mandibular width in relation to the gonion and other features near the ramus osteotomy and bone-healing site. A future study to establish a consistent definition of anatomical reference points that considers the post-IVRO healing pattern is recommended.

Facial width in patients with mandibular prognathism and facial asymmetry can be measured using a facial soft tissue scanner or CT scan. To improve the study method of using clinical images to investigate facial width, a facial scanner can provide a 3D image of facial soft tissue [6], and the use of two cameras can also provide a 3D image of the face [7]. Future studies on orthognathic surgery using 3D images and changes in facial soft tissue and bone can be expected in the case of various osteotomy procedures. Furthermore, a study that investigates various changes in the face according to proximal segment osteotomy and treatment approach is necessary.

The results of this study show that there is a difference in facial width associated with the ostectomy level of the proximal segment after orthognathic surgery using IVRO. Although a statistically significant association between facial structure and postoperative bone-healing prognosis can be expected, the masseter muscle with the mandibular ramus must be considered. The functionality of the masseter muscle affects the facial structure, and accurate prediction of the movement and direction of this muscle may be challenging [8]. A study method to identify the size and location of the facial muscles and an assessment tool, such as electromyography, may be required.

In this study, the coronal and horizontal planes were used to set the measurement points. Patients requiring orthognathic surgery have mandibular prognathism and facial asymmetry. For these patients, the reference points necessary to set a three-dimensional measurement plane are asymmetric, contributing to a distorted 3D plane and measurement errors in the preoperative CT image data. This study used three-dimensional measurement planes that are being used or can be used in clinical settings. Coronal planes with the condyle, sigmoid notch, and coronoid process were associated with the anterior and posterior lengths of the ramus. The coronoid process, sigmoid notch, and condyle are reliable reference points for measuring the mandible [9].

Researchers must consider that the horizontal length of the ramus is shortened after mandibular setback surgery with IVRO. In particular, the bony reference point in the mandible may be unreliable according to the various bone-healing patterns in the mandibular gonial angle area. Moreover, the coronal and horizontal planes of the mandible (such as the coronal and alveolar planes) may not intersect at the mandibular angle area. This occurs when the mandibular angle is more obtuse, especially in cases of severe mandibular prognathism. The current study showed numerous missing values of the mandibular bone, because the posterior inferior part of the ramus cannot be identified due to the healing patterns of mandibular ramus osteotomy, location, and/or shape of osteotomy. Therefore, there were only two measurements of bone width in this study, although there were nine measurements of soft tissue width. The use of bone width measurement is a drawback, as its use is limited in a clinical setting.

A previous study reported that the use of SSRO, a surgical approach for mandibular setback, and mandibular angle ostectomy together contributed to a decrease in the facial mandibular width [10]. Another study on the association between IVRO and facial width reported a horizontal increase in facial width after orthognathic surgery [1]. However, a previous study found that facial width temporarily increases post-IVRO and that facial soft tissue subsequently returns to the preoperative state [11]. Another study did not find a statistically significant difference in the postoperative horizontal width of the mandible between the two surgical approaches, SSRO and IVRO [2]. In addition to the study on mandibular width, a study on bone-healing patterns after SSRO and IVRO reported that the healing patterns of mandibular bone after SSRO and IVRO are similar [3]. A study on facial width after IVRO found that although the mandibular width increased immediately post-IVRO, it gradually decreased and returned to its original width within approximately 3 years. Thus, the study reported that IVRO was not associated with long-term changes in facial width [4]. In a study that compared a group of people with facial asymmetry post-IVRO and a group of people with facial symmetry, the results showed that IVRO did not lead to changes in the transverse profile of the face, thus increasing the predictability of the aesthetic results of surgery. In addition, the study found no differences in facial soft tissue and hard tissue between groups with facial symmetry and facial asymmetry as time progressed [12].

The limitations of this study were that the study used anatomical features of the mandible as measurement points, which may not correspond to the facial width determined in clinical practice. In the case of IVRO, unlike L-osteotomy, both the mandibular distal segment and coronoid process move posteriorly. The actual preoperative and postoperative locations of the coronoid process and the anteroposterior length of the ramus are different. Although the facial width remains the same, it may appear as if there is a change, depending on the anterior and posterior location of the mandible due to differences in the size depending on the perspective distance. The posteriorly relocated mandibular bone appears smaller when observed anteriorly. The distance of the posterior movement of the mandibular bone also affects its appearance. A future study is needed with the skull as the reference, or with the eye and head position fixed, and changes in facial width evaluated according to the distance between the viewer and the face when viewed anteriorly. Due to the small number of subjects in this study, it was not possible to analyze the differences according to various facial shapes. In addition, it is necessary to discuss the measurement points and methods with high reproducibility and reliability according to various face patterns for IVRO research.

The IVRO technique has undergone various modifications [13]. The facial width can be adjusted by performing osteotomy in a J-shape rather than vertical to the mandibular ramus. This study investigated the effect of ostectomy of the proximal segment of the vertical ramus osteotomy; thus, further study on facial width using modified IVRO, including that with distal mandibular segment ostectomy, is required [14].

In the case of simultaneous operation of the maxilla and mandible, when the amount of posterior maxillary impaction is large, excessive osteotomy may occur during proximal segment osteotomy at the height of the tooth occlusal surface level during IVRO, and aesthetic issues may arise due to postoperative stability, bone fusion, and the angle of the mandibular area. This study included 32 participants. Considering the various orthognathic surgeries, additional research and analysis of the surgical techniques used on the proximal segment during IVRO, targeting numerous patients at multiple facilities, would further provide information on postoperative stability, bone healing, and facial width and function.

## Conclusion

The present study demonstrated increased facial width after orthognathic surgery with IVRO, which was consistent with the results of previous studies. However, when proximal segment ostectomy is performed at the level of the mandibular tooth occlusal surface, the facial width at the height of the mandibular first molar alveolar bone and the anteroposterior location of the coronoid process may not increase significantly. In addition, the face may appear aesthetically small, considering the distance of posterior mandibular movement.

## Data Availability

Not applicable.

## Abbreviations

IVRO: Intraoral vertical ramus osteotomy
SSRO: Sagittal split ramus osteotomy
3D: Three-dimensional
CT: Computed tomography
TMJ: Temporomandibular joint
